# Medical training for universal health coverage: a review of Cuba–South Africa collaboration

**DOI:** 10.1186/s12960-020-0450-9

**Published:** 2020-02-17

**Authors:** Neil Squires, Susannah E. Colville, Kalipso Chalkidou, Shah Ebrahim

**Affiliations:** 1 Public Health England, Lincoln University, Lincoln, United Kingdom; 2 Centre for Global Health and Development, Imperial College London, London, United Kingdom; 3 London School of Hygiene & Tropical Medicine, London, United Kingdom

**Keywords:** Universal health coverage, Cuba, South Africa, Medical education

## Abstract

Achieving improvements in Universal Health Coverage will require a re-orientation of medical education towards a stronger focus on primary health care. Innovative medical curricula have been implemented in some countries, but in many low- and middle-income countries (LMICs), the emphasis remains focused on hospital and speciality services. Cuba has a long history of supporting LMICs and has made major contributions to African health care and medical training. A scheme for training South African students in Cuba was established 20 years ago and expanded more recently, with around 700 Cuban-trained graduates returning to South Africa each year from 2018 to 2022. The current strategy is to re-orientate and re-train these graduates in South African medical schools for up to 3 years as they are perceived to have inadequate skills. This negative narrative on Cuban-trained doctors in South Africa could be changed dramatically. They have highly appropriate skills in primary care and prevention and could provide much needed services to rural and urban under-served populations whilst gaining an orientation to the health problems of South Africa and strengthening their skills. Bilateral arrangements between South Africa and the United Kingdom are providing mechanisms to support such schemes. The Cuban approach to medical education may have lessons for many countries attempting to meet the challenges of Universal Health Coverage.

## Background

Demographic and epidemiological transitions coupled with increased expectations and medical innovations are placing ever greater demands on health services, often considered to be infinite [1]. Health for All [2] remains a global aspiration and has been given renewed momentum as Universal Health Coverage (UHC) is now a target of the Strategic Development Health Goals [3, 4]. Achieving UHC is likely to require systematic and radical policy change in most countries [5]. The balance of investment in health care continues to be dominated by secondary and tertiary sectors, where costs are escalating, with primary care, prevention and social care funding squeezed [6, 7].

Shortages of doctors and other health workers remain a major hurdle for achieving UHC [8], despite a decade of action launched with the 2006 World Health Report [9]. The global policy recognises that investment in the health workforce contributes to economic growth [10, 11]. The High-Level Commission on Health Employment and Economic Growth has predicted a continuing and massive growth in demand for health workers which will not be met by planned production. The impact of anticipated workforce shortages will fall disproportionately on populations already facing limited health service access.

In 2010, global leaders of health workforce education and training called for a radical transformation in medical education to meet the changing health needs of populations [12]. Doctors play an essential leadership role in achieving UHC, but in most countries, their training is orientated towards curative, high technology sub-specialisation in medicine and surgery. The financial and academic barriers to admission to medical school tend to favour the selection of students from privileged backgrounds, leading to young doctors who have limited direct experience of the health needs of poor rural and under-served urban communities, with few choosing to serve these communities [12]. Medical students still aspire to become successful in specialist medicine and develop values that are not orientated towards meeting the needs of the poor and disadvantaged in their countries [13]. Whilst there have been some interesting innovative models of health care developed to meet the needs of remote rural populations, such as rural health care development in Quebec and other models pioneered by the Training for Health Equity Network (THE Network) [14], these have yet to be replicated at scale.

The path from primary health care in the 1970s to the achievement of UHC has been defined in a WHO document [15]. Briefly, primary health care systems failed to emerge as many low- and middle-income countries (LMICs) established vertical disease control programmes, with duplication of effort and fragmentation of services. Equitable access to health care and national ability to invest resources efficiently to meet the population needs and expectations were lost. Furthermore, the Millennium Development Goals highlighted the need for strengthening primary health care. By 2010, a growing consensus emerged that primary health care was the only path to reducing waste and improving health service efficiencies, incentivising equitable quality performance, containing costs and achieving UHC [15]. Typically, in LMICs, primary health care systems are underdeveloped (or non-existent), starved of resources and have insufficient adequately trained health professionals [3]. Whilst doctors are not the only important element of a successful health care system, with the right leadership and technical skills, doctors remain an important human resource requirement for a functioning health care system [11].

## The transformation of Cuban medical education

A radical transformation in medical education occurred in Cuba following the 1955 revolution [16]. Cuba adopted an entirely different approach to medical education with the emphasis on primary health care, an interdisciplinary approach, a strong community participation, an internationalist focus, and a health workforce of sufficient size and skills to meet population needs. Much of the training is in rural polyclinics as part of a primary care team, giving students a community and primary care orientation for a large part of their training. Moreover, their values are different: solidarity with poor and disadvantaged communities, and a willingness to serve in a variety of challenging settings [17]. Importantly, Cuba has achieved impressive improvements in population health, aided by these innovations [18].

Cuba’s export and training of doctors have been equally dramatic, with over 38 000 Cuban-trained doctors working in over 60 countries of the World [19]. Cuba has trained over 100 000 doctors since the 1950s when the value of investing in health was recognised and prioritised by Castro.

In 2014, a United Kingdom–Cuba–Republic of South Africa project was established by the authors to address the role of the Cuban model of medical education in transitioning towards universal health coverage in South Africa. The findings of the project relating to the development of Cuba’s model of medical education and international engagement have been previously published [20].

This review focuses on the medical education collaboration between Cuba and the Republic of South Africa since 1994 and the policy and practical implications that have arisen. Comparisons are made with other Cuban collaborations in Africa.

## Methods

An initial meeting was held in Cuba (November 2013) with the Cuban Ministry of Health (Ministerio de Salud Publica - MINSAP) and the Cuban Pan American Health Organization (PAHO) to define plans to assess the impact of the Cuban medical education model on the delivery of care and the wider health systems in Sub-Saharan Africa (SSA), using the Republic of South Africa (RSA) as the focal country in SSA. The bilateral cooperation between Cuba and the RSA is now one of the largest Cuban international medical education initiatives. The calls for changes in medical education to meet the population needs and ensure greater equity in access to health care and health status made study of the Cuban approach of unique interest, not only in terms of how it might be adapted for use in RSA and other African countries but also what it might have to inform UK medical training.

A scoping review of the literature on Cuban medical education was conducted with support from the MINSAP, PAHO, Centro Nacional de Información de Ciencias Médicas–INFOMED and Unidad Central de Colaboración Médica (UCCM)–International Cooperation Centre. Issues of MEDICC Review (International Journal of Cuban Health Medicine) were hand searched, and a series of meetings between the research team, politicians, academics and medical students and graduates were held in Cuba, RSA and London from 2014 to 2017. Full findings of research comprising in-depth interviews with key informants, focus group discussions held with medical students and graduates, and a medical student survey of competences and career aspirations have been published [21] elsewhere, and some material is used here to illustrate the specific issues in the Cuba–South Africa programme. A PubMed search was conducted using terms relating to medical education, Cuba and Africa and was updated in April 2019. These searches generated 1909 hits of which 61 were selected based on the title and abstract. Articles providing information on Cuban medical education activities in Africa were obtained for a full assessment to find evidence of previous evaluations of Cuban medical education initiatives in Africa.

## The Cuba–South Africa collaboration

Cuba’s international medical activities following natural disasters and more recently with Ebola in West Africa are well documented [22, 23]. Less well known outside RSA is the historic agreement in 1995 between Nelson Mandela and Fidel Castro which in 1997 led to Cuban doctors deploying in RSA and the recruitment of RSA students from poor black communities moving to Cuban medical schools and training as doctors capable of meeting the health care needs of rural and under-served urban populations [24]. The selection of students from deprived communities, with the involvement of those communities, was intended to create an obligation and commitment for them to return and provide health care for these communities. The Mandela–Castro programme also aimed to tackle the historic inequalities in access to a medical education, which at that time were a largely white and middle-class preserve [25].

Despite concerns from RSA medical schools, the medical profession and an impact assessment recommending terminating the programme [26], training has increased in the last decade with much larger cohorts of around 700 RSA students annually going to study in Cuba [27]. Yet, there is a reluctance to evaluate the benefits that might be realised from this investment, with many of the key stakeholders in the programme questioning the motive of such an evaluation. It might be that the South African political backers of the scheme are concerned lest the investment be considered inefficient. Cuba is proud of its training programme which has been taken up in many African countries, and US citizens trained in Cuba have demonstrated success in examinations to work in the US health system [28–30]. Consequently, the Cubans may consider any evaluation superfluous and that it might report negatively on the Mandela–Castro programme.

Our attempts to conduct an assessment of Cuban medical education for RSA were not viewed positively, and it proved difficult to implement our plans in full. We were able to conduct stakeholder meetings in Cuba, RSA and London, and survey student and graduate competences and career aspirations using quantitative and qualitative methods. These activities are documented in non-peer-reviewed meeting reports written by the authors [31, 32]. Our assessment found that Cuban-trained students had similar self-reported confidence in conducting practical procedures and reported skills to those of RSA-trained students. The career aspirations of Cuban-trained students were focused on primary care and serving poor communities in RSA which was very different from those trained in RSA who viewed hospital careers and the possibility of emigration from RSA more positively. Valuable insights were gained from in-depth interviews:

Medicine is the same in in the world, what makes Cuba different is the constant public health references where you needed to always go back to where the problem initiated. The disease was always linked to what was happening in the community, it made us realise that closing the tap was more important than mopping the floor. (Cuban-trained doctor in RSA).

The whole program was used to fight political battles, not viewed as part of a solution for human resource improvement in our country. The government program was never internalized by our very own South African universities. Hence there is resistance in forming part of the solution (Senior Faculty member, RSA medical school).

…basically I like to help the community because I’m from a rural community so where we have little health facilities so most of the people in the community they don’t have much medical knowledge …, and I’ll be the first one in the community so, I would like to make a change. (Cuban-trained student in RSA).

The current experience of returning Cuban-trained doctors is re-education and re-orientation to the RSA system [27, 33]. It has been suggested that this approach may be insufficient and that an ‘identity perspective’ may be needed to deal with the difficult cultural readjustments for some returning trainees [33]. The Cuban education experience cannot cover practical management of HIV/AIDS (which is rare in Cuba) and does not aim to give training in conducting caesarian section (which is considered a post-graduate qualification in Cuba and the United Kingdom) [34]. Those returning currently undertake at least 1 and up to 3 years additional education in a RSA medical school. Whilst this might help ensure equivalence with RSA-trained doctors, it fails to recognise the benefits of the community orientation developed in Cuba and delays the clinical debut of those returning, potentially also undermining their commitment to return to and serve in rural areas delivering primary care.

One of the concerns about the Cuba–RSA collaboration is its cost. In the early years of Cuba’s international medical collaborations, most of the costs were borne by Cuba and not African countries. Over time, such arrangements have changed. By 2015, over 140 000 Cuban professionals were working in 67 collaborating countries. Cuba covers all costs for 20 countries; in 17 countries, costs are shared and the other 30 countries pay Cuba for their medical support [35].

The potential of RSA’s investment in Cuba to deliver both health benefits and contribute to social transformation and economic benefit for previously marginalised populations remains to be explored. As President Zuma was one of the High-Level Commissioners on the Health Employment and Economic Growth Commission [10, 11], it might be expected that RSA would realise the benefit from such investments. However, this is difficult because the health system in RSA is speciality-dominated, remains inequitable with maldistribution of resources and is fragmented [36]. Many challenges exist: workforce shortages, emigration of doctors and skills-mix imbalances. The private health sector is buoyant, catering for the wealthy minority of less than 20%, but spending about 60% of the 8.4% of GDP spent on health [36]. Access to and quality of health care in rural and disadvantaged urban populations remain a serious problem. There is growing evidence that medical schools that are socially orientated and accountable to local communities—in line with the Cuban approach—can produce doctors who have a different outlook to those trained in traditional systems [37]. For example, students recruited from disadvantaged rural communities to socially accountable medical schools are more likely to indicate wanting to practise in rural areas and not emigrate [38, 39].

UHC is talked about, but there remain insurmountable challenges of quality, access and affordability. A National Health Insurance Policy [25] is being rolled out as a means of achieving UHC, but the human resources for this are very limited. The Mandela–Castro programme is acknowledged as one of a number of strategies to increase the production of health professionals. RSA’s human resources for health strategy [40] also emphasise re-orientation towards public health and primary health care to meet the health needs of poor South Africans.

RSA’s long heritage of British medical education traditions and academic excellence, selection of the brightest students and its strong emphasis on specialisation have not helped in developing primary health care. In the last two decades, steps have been made to focus on primary health care, move teaching out of hospitals and into communities and to improve access for black and disadvantaged students [41–43]. Medical schools have been established outside of the major cities and provide education for predominantly black students. This has resulted in the proportion of black, mixed-race and Indian medical students in RSA medical schools increasing to two thirds of the annual intake to one third of white students [41].

## Comparisons with other Cuban collaborations

Long-standing relationships between several African countries and Cuba were based initially on military support for achieving political independence from colonial powers. Cuban medical professionals often worked alongside the military brigades and remained in the country to support later phases of health service development and medical training [44]. Table 1 shows details of the types of collaboration. Most involved both the deployment of Cuban health professionals in African countries, the training of Africans in Cuba and the establishment of medical schools in Africa, initially with Cuban academics providing the training. The scale of Cuba’s engagement with the provision of medical care and training of doctors in Africa is large. Table 2, derived from World Health Organization Global Health Observatory and Cuban sources, shows the African countries that have received Cuban support, the population, number of doctors and hospital beds per 10 000 population, numbers of Cuban doctors based in African countries in 2016. Data on the cumulative number of consultations and surgical operations conducted from initial Cuban involvement up to the end of March 2019 are available [61] but cannot be used to estimate annual or workload per Cuban doctor. Data on surgical procedures in African countries are being collected but no country-level data are publicly available. However, the population estimates for the most recent year available, doctors per 10 000 population, hospital beds per 10 000 population and ratio of Cuban to African doctors provide some context. There are limits to what can be understood from these data: (a) Cuban doctors have served in a large number of African countries; (b) the proportion of Cuban to African doctors varies markedly, from less than 1% to a quarter of the medical workforce in smaller African countries where they play a major role in medical education; (c) African countries receiving Cuban medical support have wide differences in their medical health workforce and hospital bed facilities.

**Table 1:** African countries and Cuban health collaboration

Country (date of the first collaboration)	Type of collaboration	Description	Citation
Algeria, 1962	Cuban military and health brigades First medical mission to Africa, 1963	In this first health brigade, it was not clear who would pay. After interventions by Che Guevara, the Cubans paid.	[45, 46]
Angola, 1975	Cuban military and health brigades Health professionals training in Angola Medical training in Cuba	Creation and implementation of medical training programmes in rural areas. The programme, developed over a period of 6 years and based on a model tried and tested by the ELAM, is implemented mainly by Cuban professors	[47]
Cape Verde, 1975	Cuban brigades to provide health care following independence Medical training in Cuba	Cape Verde had no medical school until 2015 and was dependent on international collaborations. Human resource secondary data were used to map the training of doctors. Cuba has trained almost half (189) of the doctors trained since 1975.	[48]
Gambia, 1999	Cuban brigades Medical training in Cuba Establishment of medical school in the Gambia with Cuban support (1999) Development of community-based medical training (2006)	Demonstrates the evolution of independence from the United Kingdom (1965) to establishing a community-based medical training that focuses on recruiting students from rural areas. Long-term Cuban and Gambian government relationship critical to the success of the programme.	[49, 50]
Guinea-Bissau, 1966	Cuban military and medical brigades (1966) University scholarships to Cuba 1972–1974 Establishment of medical school (1986) staffed by Cuban academics and interns G-B students sent to Cuba for training (1998–2006)	Medical school closed in 1998 due to conflict and was re-opened with Cuban support in 2006. Until 2013, medical school was staffed exclusively by Cubans. From 2012, G-B took over all costs of the programme.	[51, 52]
Guinea Equatorial, 2000	Establishment of medical school (2000) Medical training in Cuba (sixth year only)	Medical students from Equatorial Guinea Bata Medical School are taught by Cuban academics and go to Cuba to take their sixth academic year and graduate. So far, 208 doctors have graduated from the Bata school.	[53, 54]
Mozambique, 1975	Cuban military and medical brigades following independence (1975). Cuban doctors contracted to work in Mozambique from 1992 to 2000 under a pooling project. Cuban academics recruited to implement a reformed curriculum since 2003.	Long-standing health collaboration. Pooling project established with international funds (following fall of USSR) from Switzerland, Netherlands and Norway. Field hospital and medical staff sent following recent (2019) cyclone. Currently, nearly 400 Cuban doctors working in the country.	[55, 56]

**Table 2:** Table caption

Country	Population	Doctors/10000	Beds/10000	Cuban Doctors 2016	African Doctors	Percent Cuban/African
Algeria	42,228,000	18.30	19	347	77277	0.4
Angola^a^	30,809,000	2.15	8	815	6624	12.3
Botswana	2,254,000	3.69	18	50	832	6.0
Burkina Fasso	19,751,000	0.60	4	19	1185	1.6
Burundi	11,175,000	0.50	8	~	559	~
Cape Verde^a^	543,000	7.69	21	41	418	9.8
Chad	15,477,000	0.47	4	7	727	1.0
Congo	84,068,000	1.16	16	28	9752	0.3
Djibouti	958,000	2.20	14	26	211	12.3
Ethiopia	109,224,000	1.00	3	~	10922	~
Gabon	2,119,000	3.61	13	30	765	3.9
Gambia^a^	2,280,000	1.07	11	64	244	26.2
Ghana	29,767,000	1.28	9	~	3810	~
Guinea Bissau^a^	1,874,000	2.00	10	26	375	6.9
Guinea Conakry	12,414,000	0.79	3	11	981	1.1
Guinea Ecuatorial^a^	1,308,000	4.00	21	135	523	25.8
Kenya	51,393,000	1.99	14	~	10227	~
Lesotho	2,108,000	0.68	13	~	143	~
Mali	19,077,000	1.39	1	~	2652	~
Mauritania	4,403,000	1.65	4	~	726	~
Mozambique	29,495,000	0.55	7	221	1622	13.6
Namibia	2,448,000	3.72	27	55	911	6.0
Niger	22,442,000	0.50	3	4	1122	0.4
Nigeria	195,874,000	3.83	≈	~	75020	~
RASD	513,000	≈	≈	~	~	~
Rwanda	12,301,000	1.40	16	~	1722	~
San Tomé Príncipe^a^	211,000	3.20	29	9	68	13.3
Seychelles	96,000	9.46	36	~	91	~
Sierra Leona	7,650,000	0.25	4	~	191	~
South Africa	57,779,000	8.02	28	337	46339	0.7
Swaziland	1,357,000	≈	21	18	~	~
Tanzania^a^	56,318,000	≈	≈	26	~	~
Uganda	42,723,000	0.91	5	4	3888	0.1
Yemen	28,498,000	3.10	7	~	8834	~
Zimbabwe	14,439,000	0.76	17	25	1097	2.3

Sources: -http://cubacoopera.uccm.sld.cu/datos-y-estadisticas/indicadores-de-servicio/?print=pdfIndicadores de Servicios en las Brigadas Médicas en el Exterior acumulados hasta el 31 de marzo de 2019 Data on number of doctors from: http://en.granma.cu/mundo/2016-07-15/cubas-international-health-cooperationPopulation data from -https://data.worldbank.org/indicator/sp.pop.totlHospital beds/10000 from Global Health Observatory data repository http://apps.who.int/gho/data/node.countryDoctor/10000 from 2017 update, Global Health Workforce Statistics, World Health Organization, Geneva

*RASD*Sahrawi Arab Democratic Republic (formerly Western Sahara)

^a^involved in medical training

~ no Cuban doctors in Country at 20163

≈ no data available

*Involved in medical training ~ no Cuban doctors in the country at 2016 ≈ no data available. Sources: [57] and Doctor/10000 from 2017 update, Global Health Workforce Statistics, World Health Organization, Geneva [60]

Similar but smaller Cuban collaborations between Pacific Islands have resulted in rapid increases in improvements in health services and the medical training of Pacific Islanders in Cuba, the majority of whom return home to contribute to the health system. These schemes have been welcomed and provide inexpensive means of medical training for small countries in which it would be difficult to create a viable medical school. Despite the number of schemes that have been set up, it has been noted that mechanisms to document their evolution and to enable assessment of the impact on health outcomes have not yet been established [62]

Collaborations involving large scale deployment of Cuban health professionals have occurred in Latin America to provide health care for remote and under-served communities. For example, in Brazil, the government attempts to improve the maldistribution of health professionals by financial incentives proved insufficient and led to the Más Medicos programme. This collaboration with Cuba contracted over 14 000 Cubans to work in Brazil and was considered a major success [63, 64]. However, it was not popular with Brazilian doctors and some politicians. The programme was challenged by a joint lawsuit by the Brazilian Medical Association and Federal Council of Medicine to the Federal Supreme Court claiming employing Cuban doctors was illegal [65]. Finally, in 2018, the programme was abandoned with doctors returning to Cuba following negative remarks by the new Brazilian government [66].

The recently published Lancet Commission on the future of health and health care in Sub-Saharan Africa covered the colonial and post-colonial history of health care in Africa, but made no mention of the Cuban collaborations that have and continue to contribute to achieving UHC, even in the most affluent African country—the RSA [67]. This oversight may have occurred as the Commission was ‘prompted by Sub-Saharan Africa’s potential to improve health on its own terms, and largely with its own resources’. The Commission noted that only 9% of the graduates from African medical schools work in rural general practice whereas 22% migrate outside of Africa—a situation that does not give optimism for UHC in Africa.

## The opportunity for the Republic of South Africa and the United Kingdom

Many factors are now aligned to make RSA ripe for a bold experiment to re-engineer primary care through a radical transformation of medical education. With the prospect of around 700 Cuban-trained South Africans returning to RSA each year from 2018 to 2022, a strategy for how to make effective use of this cohort of primary care orientated clinicians is developing. Returning Cuban-trained doctors could help achieve UHC if their value was recognised and current approaches to their integration changed. However, for the past 2 years, sufficient posts for newly graduated RSA doctors in the public sector were not available. For example, in Kwa-Zulu Natal, only 2126 of the 3191 posts available for medical practitioners were filled in 2017 due to shortages of funding for posts [68].

The current negative narrative on Cuban-trained doctors in RSA can be changed. The United Kingdom has strong links with RSA and has been the beneficiary of RSA-trained doctors as a receiving country of the brain drain of RSA expertise. The United Kingdom’s current Development Strategy (November 2015) was titled ‘Tackling Global Challenges in the National Interest’ [69], recognising that the UK Aid programme can bring mutual benefits supporting priorities at home as well as development abroad. Health Education England (HEE) supports a programme called the Africa Placement Scheme deploying GP trainees as Global Health Fellows for a year to work in rural RSA clinical settings, with salaries paid by RSA. This gives rich clinical and management experience to doctors who return to the United Kingdom, as well as addressing the clinical needs in RSA. In addition, HEE has established ‘Improving Global Health’ Fellowships which enable National Health Service staff to volunteer for 6-month placements running quality improvement programmes in RSA. This programme is an example of mutual interest in action—with RSA’s needs being met whilst delivering benefits for the United Kingdom in terms of increased recruitment of doctors to GP training and a powerful education and leadership opportunity for those who take up the challenge.

There is now an opportunity to develop this scheme further. The RSA government is exploring UK support for a mentoring programme for Cuban-trained RSA doctors returning from Cuba. UK trainees could be used to supervise and train cohorts of returned doctors, both through online communities of practice during their final year—ahead of the Global Health Fellow arriving—and in-country providing them with the required skills and competencies to deliver safe and effective primary health care.

Placements could be delivered in rural areas, where Cuban-trained RSA doctors are working in community service positions. Through HEE’s Global Health Fellowship Programme, UK Global Health Fellows can be provided with support to mentor these doctors as they work to address identified competency gaps. This approach fits with the original intent of the Mandela–Castro programme which selected students from rural communities where they would return to work. UK trainees would also be provided with mentoring support for their training role by senior UK GP trainers and would build their training competencies working alongside RSA peers. Additionally, co-locating Improving Global Health volunteers at the same rural sites as Global Health Fellows would support this programme through both delivering quality improvement projects during their 6-month placements and building the local capacity to use quality improvement methodologies on-site.

## Conclusions

RSA has created an opportunity to radically transform primary care delivery, making more effective use of the investment it has made in training its students in Cuba. The proposed collaboration between the RSA and the United Kingdom to support the development of primary care could benefit both the United Kingdom and RSA, through mutual learning and exchange. In RSA, these benefits would be in the improvement of rural primary health care and the development of an improved medical workforce in rural areas. Policy recommendations for sustainable human resource development for health in RSA and with some relevance to other countries are shown in Table 3.

**Table 3:** Policy recommendations for sustainable human resource development for health

• Global policies for Universal Health Coverage require innovative ways of tackling problems of maldistribution of doctors and migration from low- and middle-income countries.	
• The South Africa–Cuba cooperation to train black, disadvantaged South African students in Cuba to become doctors to provide services to rural and urban under-served populations in South Africa provides a major opportunity for Universal Health Coverage.	
• The current negative narrative about these young doctors insufficiently skilled for South African medicine and requiring years of further training in South African medical schools has to be changed. These Cuban-trained doctors have excellent skills in primary care, prevention and teamwork which are of great value to strengthen primary health care services.	
• Bilateral arrangements between South Africa and the United Kingdom may provide mechanisms to support rural primary health care training schemes.	
• The Cuban approach to medical education may have lessons for many countries attempting to meet the challenges of Universal Health Coverage.	

In the UK National Health Service, volunteering to support capacity building in RSA could have an impact on retention, helping to reduce the current challenge of trained and experienced staff leaving the NHS. Further, the desire to have career fluidity and to view themselves as global citizens have been identified as key character traits of millennials and those in generation Z, meaning that supporting international volunteering for staff spanning these age groups may have an even greater effect on efforts to retain these health workers. The benefits of promoting overseas volunteering for the NHS and globally have been reviewed by the All-Party Parliamentary Group on Global Health [70].

There are also other potential benefits for the United Kingdom linked to developing training to equip doctors to work in primary care in under-served areas, as this remains a challenge in the United Kingdom. Collaboration with some of the newly created UK medical schools and those established schools which are seeking to increase the training of health workers orientated to working in primary health care could be a further benefit of the learning gained from the United Kingdom collaborating with RSA and Cuba.

## Data Availability

The datasets used and/or analysed during the current study are available from the corresponding author on reasonable request.

## Abbreviations

HEE: Health Education England
LMIC: Low- and middle-income countries
RSA: Republic of South Africa
UHC: Universal Health Coverage
